# The Emerging Clinical Role of Spermine in Prostate Cancer

**DOI:** 10.3390/ijms22094382

**Published:** 2021-04-22

**Authors:** Qiang Peng, Christine Yim-Ping Wong, Isabella Wai-yin Cheuk, Jeremy Yuen-Chun Teoh, Peter Ka-Fung Chiu, Chi-Fai Ng

**Affiliations:** SH Ho Urology Centre, Department of Surgery, The Chinese University of Hong Kong, Hong Kong, China; pengqiang@surgery.cuhk.edu.hk (Q.P.); christinewong@surgery.cuhk.edu.hk (C.Y.-P.W.); isabellacheuk@surgery.cuhk.edu.hk (I.W.-y.C.); jeremyteoh@surgery.cuhk.edu.hk (J.Y.-C.T.)

**Keywords:** prostate cancer, spermine, polyamine, cancer metabolism, biomarker

## Abstract

Spermine, a member of polyamines, exists in all organisms and is essential for normal cell growth and function. It is highly expressed in the prostate compared with other organs and is detectable in urine, tissue, expressed prostatic secretions, and erythrocyte. A significant reduction of spermine level was observed in prostate cancer (PCa) tissue compared with benign prostate tissue, and the level of urinary spermine was also significantly lower in men with PCa. Decreased spermine level may be used as an indicator of malignant phenotype transformation from normal to malignant tissue in prostate. Studies targeting polyamines and key rate-limiting enzymes associated with spermine metabolism as a tool for PCa therapy and chemoprevention have been conducted with various polyamine biosynthesis inhibitors and polyamine analogues. The mechanism between spermine and PCa development are possibly related to the regulation of polyamine metabolism, cancer-driving pathways, oxidative stress, anticancer immunosurveillance, and apoptosis regulation. Although the specific mechanism of spermine in PCa development is still unclear, ongoing research in spermine metabolism and its association with PCa pathophysiology opens up new opportunities in the diagnostic and therapeutic roles of spermine in PCa management.

## 1. Introduction

The polyamines putrescine, spermidine, and spermine are small polycations derived from amino acids and exist in all organisms; they are essential for normal cell growth and function [1]. Cancer cells show different phenotypes from normal cells, and these distinct phenotypes translate into profound metabolic changes, which are crucial for the development and progression of cancer [2].

In the prostate, polyamine metabolism is important since it synthesizes and accumulates the highest levels of polyamines, particularly spermine. As one of the polyamine members, spermine was first reported in human semen as early as the 17th century by Antoni van Leeuwenhoek [3]. High secretory activity of the luminal epithelial cells surrounding the glandular ducts maintains a relatively high spermine level in the secretory fluid of a healthy prostate. An early study by Harrison [4] found that the human prostate gland synthesizes the highest levels of spermine per day, with an average of 130 mg/100 g, which is significantly higher than pancreas tissue that produce the second highest level of spermine per day (16 mg/100g). The purpose of this high synthesis rate is to excrete it into prostatic fluid, making the prostate the only human tissue where the largest proportion of its synthesized polyamines are primarily intended for exporting instead of supporting cell proliferation [5].

In the early stage of PCa, most cases are organ-confined and grow slowly without symptoms [6]. A significant association was found between urine/tissue spermine levels and the degree of tumor malignancy in PCa patients. Normal and benign prostatic hyperplasia (BPH) tissues have a high spermine content, whereas with the development of PCa, the changes in normal glandular morphology or cell metabolism resulted in decreased spermine level, especially in those with metastatic PCa tissues. Hence, a dramatic reduction in prostatic spermine content could indicate the conversion of prostatic tissue from a benign to a malignant phenotype [7]. In addition, Smith et al. [8] reported that spermine inhibited the growth of PCa both in vivo and in vitro. However, the effect of spermine on PCa varied; it inhibited the growth of rat PCa cells with low metastatic potential, but not those PCa cells with highly metastatic potential, indicating a shift of spermine response from sensitivity to resistance as tumor progressed to a metastatic state. Koike et al. further found that the lack of antizyme induction correlates with spermine resistance in the prostate [9].

Although metastatic PCa is incurable [10], the progression from local PCa to metastatic PCa often takes a long time; therefore, it is potentially curable if it is diagnosed and treated early. In a study based on four public data sets by Rhodes et al., a set of genes involved in polyamine metabolic pathways was found to be consistently and significantly dysregulated in PCa [11]. Therefore, the high polyamine levels in the prostate, mainly spermine, may help us discover new biomarkers with high sensitivity to detect aggressive PCa and develop new therapies.

## 2. The Sources of Spermine

### 2.1. Extracellular Sources

The body pool of spermine is maintained by three sources: food consumption, intestinal microbiota, and endogenous (de novo) biosynthesis arising from increased synthesis enzymes activity [12]. This external dietary source provides a larger quantity of spermine than the endogenous biosynthesis, and a wide range of foods derived from plants and animal tissues in our diet contain high spermine. Spermine contents are typically high in the internal organs and meat of warm-blooded animals and soybeans, whereas high levels of putrescine and spermidine are found in fish flesh, fruits, and vegetables [13]. Spermine-rich foods can be absorbed from the intestinal lumen and distributed in the body through systemic circulation, increasing its content in multiple systems [14]. Different dietary habits in different regions varied in polyamine intakes amount and composition. A study comparing dietary polyamines intake in six West European countries, the USA, and Japan showed that the Western-style diet had high polyamine intake, whereas the Japanese diet represented a significantly lower polyamine intake, especially in spermine [13].

### 2.2. Endogenous Biosynthesis

**Figure 1:**
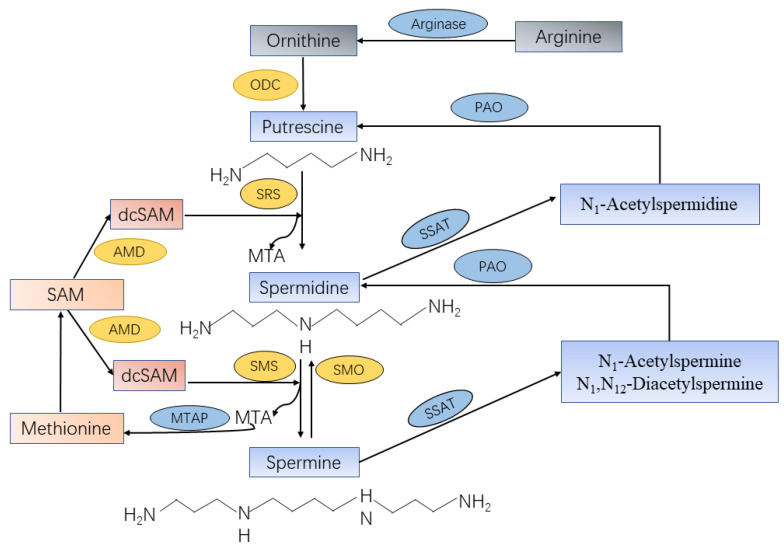
Spermine synthesis and catabolism in mammalian cells. Arginine produces ornithine through the action of arginase. Ornithine decarboxylase (ODC) is the first rate-limiting enzyme in spermine synthesis, in which ornithine is decarboxylated to produce putrescine. Spermidine synthase (SRS) and spermine synthase (SMS) are constitutively expressed aminopropyltransferases that catalyze the transfer of the aminopropyl group from decarboxylated S-adenosylmethionine (dcSAM) to putrescine and spermidine to form spermidine and spermine, respectively, as well as methylthioadenosine (MTA). The dcSAM is converted from S-adenosylmethionine (SAM) by enzymatic role of adenosylmethionine decarboxylase (AMD) as a second rate-limiting enzyme in polyamine synthesis. The central enzyme in the polyamine catabolic pathway is spermidine-spermine-N1-acetyltransferase (SSAT), which monoacetylates spermidine and mono-/diacetylates spermine. These acetylated polyamines are the substrates of N1-acetylpolyamine oxidase (PAO), which are catalyzed to form putrescine or spermidine, respectively. Spermine can be oxidized directly and specifically to produce spermidine by spermine oxidase (SMO).

De novo biosynthesis of spermine in vivo mainly triggered by amino acids such as ornithine, methionine, and arginine (Figure 1). The pathway starts with the production of ornithine from arginine by the action of arginase. Ornithine is then decarboxylated to produce putrescine with the involvement of ornithine decarboxylase (ODC), a key rate-limiting enzyme in the first step of polyamine synthesis. Putrescine can generate spermidine and methylthioadenosine (MTA) with sequential reactions of aminopropyl group from decarboxylated S-adenosylmethionine (dcSAM) under the action of spermidine synthase (SRS). The dcSAM is converted from S-adenosylmethionine (SAM) by enzymatic role of adenosylmethionine decarboxylase (AMD) as a second rate-limiting enzyme in polyamine synthesis. Then, the biosynthesized spermidine can be sequentially converted into spermine and an additional MTA by spermine synthase (SMS) with sequential reactions of the secondary dcSAM molecule [15]. The reactions to form spermine and spermidine are irreversible, but the mutual conversion of polyamines can occur through the action of spermine oxidase (SMO) or N1-acetylpolyamine oxidase (PAO) after acetylation [16]. Spermine can also be oxidized directly to form spermidine, H_2_O_2_, and 3-aminopropanal.

The catabolic mechanism of spermine contains two steps that are catalyzed by rate-limiting catabolic enzymes spermidine/spermine N1-acetyltransferase (SSAT) and PAO. SSAT plays an important role in polyamine homeostasis as a propylamine acetyltransferase that convert spermine to monoacetylated metabolites (N_1_-acetylspermine) or diacetylated spermine (N_1_, N_12_-diacetylspermine) [17]. These acetylated spermine have at least two potential fates, either export from the cell by transporter or as PAO substrates. PAO, a constitutively expressed peroxisomal polyamine oxidase, generates putrescine or spermidine from acetylated spermine, with the production of H_2_O_2_ and 3-acetyaminopropanal [16]. Both SMO and PAO have the potential to generate substantial amounts of reactive oxygen species (ROS), resulting in oxidative damage [18]. In addition, online database cBioPortal [19] and GEPIA [20] were used to further investigate whether those enzymes that were directly related to spermine synthesis were dysregulated in PCa. Results revealed that the transcription level of SMS, SMO, and SAT1 were upregulated, whereas AMD1 expression level had no change in PCa tissues compared with normal. Furthermore, less than 1% mutation level of SMS and AMD1 in PCa were observed, but SMO and SAT1 had no alterations in PCa.

## 3. Molecular Mechanisms of Spermine for PCa Carcinogenesis and Progression

### 3.1. Polyamine Metabolism, Oncogenes and Tumor Suppressors

A variety of oncogenes, including Myc, Ras, and PI3K-mTOR, etc., have been reported to target polyamine metabolites, despite their molecular regulatory mechanism is not fully understood. ODC was reported to be a transcription target of the Myc oncogene, and elevated Myc expression leads to increased ODC mRNA and protein that are necessary to drive prostate cell proliferation and malignant transformation [21]. Except for Myc, other oncogenes were also identified to be associated with enzymes of spermine metabolism. Upon Ras activation, ODC is also induced, and studies further confirmed that ODC expression is controlled by the Ras effector pathways [22,23]. As for the polyamine catabolism pathway, Ras can interfere with the transcriptional activation of SSAT by peroxisome proliferator-activated receptor-γ (PPARγ), leading to the downregulation of SSAT, thereby maintaining the elevated spermine levels in transformed cells [24]. Amaia et al. found that increased AMD enzyme occurs frequently in PTEN-deficient PCa cells, and treatment with the mTORC1 inhibitor resulted in a loss of AMD protein. Further research revealed that activated mTOR can indirectly block the proteasomal degradation of pro-AMD and stabilize pro-AMD through phosphorylation at residue S298, leading to increased AMD and spermine biosynthesis in PCa [25]. Another encouraging finding includes the potential of targeting the methionine salvage pathway such that a high level of MTAP in PCa is maintained. A combinational strategy targeting MTAP and accelerating polyamine catabolism has a synergistic inhibitory effect on androgen-sensitive and castration refractory PCa models in vitro and in vivo [26].

Compared with the numerous reports on the interaction between polyamine metabolism and oncogenes, there are few reports on the relationship between polyamine metabolism and tumor suppressor genes. p53 is a potent tumor suppressor, and its mutation is a central element in the initiation and progression in at least half of all human cancers, including PCa [27]. As a transcription target of p53, SSAT was activated by p53 to induce lipid peroxidation and ferroptosis in response to ROS-induced cell stress, which lead to tumor growth suppression. Therefore, the tumor-suppressive function of p53 appears to be partly caused by the direct transcriptional activation of SSAT and SSAT-dependent lipid peroxidation and ferroptosis [28].

### 3.2. Polyamine Metabolism in PCa-Associated Oxidative Stress and Inflammation

ROS, such as hydroxyl radical, superoxide, and hydrogen peroxide, may contribute to the initiation and development of PCa, as well as the conversion of PCa into castration refractory PCa, via regulation of androgen receptor (AR) signaling [29]. In human, NKX3.1 has been proposed to be an essential factor in PCa carcinogenesis, which interacts with AR to promote PCa cell viability [30,31]. NKX3.1 can either by direct modulation of gene targets or by indirect regulation of AR expression to prevent cell apoptosis. Meanwhile, activated AR-JunD complex can induce SSAT expression, which in turn initiates enhanced polyamine oxidation that produces high ROS levels in certain PCa cells [32]. ROS generated by the activation of spermidine/spermine oxidation pathway can activate more than one mechanism to help androgen-dependent PCa cell survival and promote castrate-resistant PCa growth, as well as its metastasis. As prostatic epithelia produce a large excess of spermine, androgen-induced SSAT gene expression causes spermine oxidation and H_2_O_2_ production, which could be a major reason for the high ROS levels in the prostate epithelia, and further imply that spermine catabolism may be a potential source of cancer initiation [33]. Moreover, Pegg’s study revealed that the oxidation products derived from spermine oxidation may be exacerbated by a fall in spermine, since spermine has an antioxidant effect as a free radical scavenger [18].

Although the ROS production in the polyamine metabolic pathway mainly comes from SMO and SSAT/PAO, Pledgie et al. found that the majority of damaging ROS was mainly from SMO. This suggested a pathway linking inflammation to carcinogenesis through polyamine catabolism in general, which may be mediated by SMO specifically [34]. Goodwin et al. verified that SMO staining was higher in the cancer regions of prostate tissues, especially in the prostatic intraepithelial neoplasia (PIN) compared with normal tissues, indicating that enhanced SMO expression was an early event of PCa. Thus, increased H_2_O_2_ resulting from elevated SMO confirmed a molecular link between inflammation and carcinogenesis in PCa [35]. There was also evidence indicating that the use of aspirin was inversely linked to the risk of developing PCa [36]. The responses of PCa to aspirin were varied, and the shift from resistance to sensitivity was associated with decreased SSAT activity, which provides in vitro evidence that the sensitivity of human PCa cells to aspirin was correlated with cellular SSAT activity status [37]. Aspirin is expected to decrease SSAT activity in early PCa, thereby increasing tumor cell sensitivity to aspirin and ultimately suppressing tumor growth by altering cellular polyamine content.

### 3.3. Anticancer Immunosurveillance

Polyamines are involved in the establishment of an immunosuppressive tumor microenvironment and as a reason for the failure of immunotherapy [38]. A polyamine-blocking therapy (PBT) combining the DFMO with a novel polyamine transport inhibitor inhibited tumor growth and promoted durable protection against tumor recurrence in immunocompetent mice, but not in T-cell-deficient athymic nude mice [39]. A study by Alexander confirmed that the antitumor effect of PBT was T-cell dependent, accompanied by increased T-cells and decreased immunosuppressive tumor infiltrating cells. This provided a novel strategy to suppress tumor growth and reverse tumor immunosuppression by targeting polyamines [40]. Increased polyamine uptake by immune cells resulted in decreased cytokine and antitumor immune molecules required for antitumor activities. Thus, in an environment with increased polyamine, immune cells may lose their antitumor immune functions that facilitate cancer cell invade and metastasize [41].

Spermine as an inhibitor of immune responses has been widely reported. For example, spermine inhibits the generation of nitric oxide in bacterial endotoxin-activated macrophages [42], proinflammatory cytokine synthesis [43] and macrophage activation [44] and show immunosuppressive to T cells in vitro and in vivo [45]. A recent study by Singh et al. showed that ODC could regulate the activation of M1 macrophages, and its deletion could lead to enhanced M1 expression that promote tumor-killing responses [46]. These findings indicated that polyamine, especially spermine, played a regulatory role as an immunosuppressive effector by targeting T cells and suppressive myeloid cells, providing a survival mechanism and restored a more favorable tumor microenvironment to escape tumor immune response.

### 3.4. Apoptosis

Apoptosis suppression is thought to play a central role in the development and progression of cancer. A study by Peggger et al. indicated that decreased ODC activity and polyamine levels after castration induced apoptosis in prostate epithelial cells [47], which imply a protective effect of spermine on apoptosis. Moreover, inhibiting polyamine catabolism by suppressing key polyamine catabolism enzymes could prevent cyclin-dependent kinase inhibitor-induced apoptosis in PCa [48]. Some research groups also revealed that the death of PCa cells induced by some polyamine analogues were actually based on their ability to induce apoptotic cell death, which was in line with the merging evidence that polyamines are actively involved in apoptotic cell death [49,50]. BIS, a novel spermine analogue, induced apoptosis and improved radiosensitivity in human PCa cell lines and xenografts nude mice [51]. However, a study by Mi et al. tested that analogue BE-3-3-3 treatment prevented the growth of PCa cell lines without activation of the apoptosis pathway [5]. As a structural analog of spermine, it is possible that BE-3-3-3 might still mimic protective behavior of spermine. The effect of spermine biosynthetic enzyme AMD inhibitor on the process of apoptotic cell death further support the notion that increased spermine level plays a role in cellular resistance to apoptotic cell death [52]. The nature of the protective effect of spermine is not well known, but several mechanisms, such as endonuclease inhibition [53], DNA stabilization [54], and DNA defense against oxidative stress [55] have been proposed. To conclude, spermine is involved in PCa cell apoptosis, which makes it ideal target for PCa therapeutic action.

## 4. Spermine as a Biomarker for PCa

A literature search was performed on several English databases (Pubmed, Embase, Cochrane library, Scopus, Web of Science) using the Medical Subject Heading (MeSH) terms and free text words as a combination of strategy, including “prostatic neoplasms”, “prostate neoplasm”, “prostate carcinoma”, “prostate cancer”, “prostate cancers”, “cancer of the prostate”, “prostatic cancer”, and “spermine”. (as of February 16, 2021). A total of 167 published original and review articles were identified through literature search and manual search of citations from identified articles and selected journals. Among these articles, twenty-four articles focused on the detection of spermine level in PCa from urine, tissues, expressed prostatic secretions (EPS), and erythrocyte were identified, and their characteristic are summarized in Table 1.

### 4.1. Urine

As early as the mid-1970s, Sanford et al. discovered that the excretion of polyamines in the urine of patients harboring PCa was higher than normal individuals [56]. In the same year, Fair et al. reported a significant elevation of urinary spermidine content by Spectronic 20 colorimeter in PCa patients, but not putrescine and spermine [57]. After that, there were scattered reports about the role of spermine and polyamines in the urine of PCa patients. As shown in Figure 1, although spermine is mainly excreted into urine in the form of the monoacetyl derivative of spermine, the diacetylated derivatives of spermine (DiAcSpm) had lower secretion level in urine but with less variation in the population, thus may be a more reliable biomarker. In 1995, Sugimoto et al. found that DiAcSpm was significantly elevated in malignant tumors of the genitourinary system, including PCa, using high-performance liquid chromatography (HPLC) [58]. Soon after, a study by Hiramatsu et al. further revealed that decreased urinary DiAcSpm level occurred in PCa patients with effective treatment, and its elevation was correlated with poor prognosis and recurrence [59]. After that, there was no further report on urinary spermine in PCa patients.

A pilot study conducted in Hong Kong [60] using ultraperformance liquid chromatography–tandem mass spectrometry (UPLC–MS/MS) found a significantly decreased urine spermine content in PCa patients, showing its potential as a novel noninvasive diagnostic biomarker that can help distinguish PCa from non-cancerous cases including BPH. A large-scale validation study by Chiu et al. showed that in men with an elevated PSA level of 4.0–20.0 ng/mL, urine spermine can act as a noninvasive test to identify men at a higher risk of high grade PCa (HGPCa). The urine spermine and a multivariate Spermine Risk Score (combining urine spermine, prostate volume, PSA, and digital rectal examination) could act as a guide to predict PCa and HGPCa. Using the urine Spermine Risk Score, a negative predictive value of 95% for HGPCa was achieved and 37% unnecessary biopsies could be avoided [61].

Urinary spermine level was found to be significantly reduced in PCa and could serve as a potential biomarker for noninvasive diagnosis of PCa. Although the observation of such an elevated DiAcSpm level in PCa urine was inconsistent with spermine, it is in line with results of previous literature about PCa studies. Increased SMO and SSAT expression were well reported in the early PIN and cancer regions of PCa patients, which resulted in a depletion of spermine content [35,62]. This also supported the observation of increased urinary DiAcSpm content in PCa, which resulted from the enzymatic action of SSAT converting spermine to DiAcSpm (Figure 1).

### 4.2. Tissue

Polyamine measurements in PCa cell lines with different degree of differentiation revealed that poorly differentiated cell lines contained lower spermine concentrations [63]. A similar correlation was found between tissue spermine level and degree of differentiation in human PCa tissue, as well as in the urine of PCa patients [7,60,64,65] (Table 1). Metabolic studies tools such as proton magnetic resonance spectroscopy (^1^H-MRS) or high-resolution magic-angle spinning (HRMAS) have been used to evaluate metabolic information in the prostate, which was obtained noninvasively from multiple, distinct regions of prostatic tissue in situ or from intact biopsy material. In 2000, Graaf et al. found through HPLC that normal and BPH tissues had higher levels of spermine, whereas in tumor tissues, especially those metastatic PCa tissues, spermine levels were significantly lower, which might serve as a biomarker for malignant prostate tissues [7]. Swanson et al. also demonstrated that the content of spermine in PCa was significantly reduced or absent in 80% of higher-grade (Gleason Score ≥7) PCa samples using HRMAS, which indicated that spermine reduction is not only an early indicator of PCa development, but also an indicator of PCa aggressiveness [64]. These results were also supported by other studies, which confirmed that spermine concentration was significantly lower in PCa tissues than healthy glandular tissues [66] and can be used as a marker to assess PCa aggressiveness, in terms of cancer Gleason scores [65] and stages [67]. Maxeiner et al. had tried to assess whether spermine level could be used to predict PCa recurrence, forty-eight PCa cases were divided into 3 groups according to their clinical and pathological information, as well as their biochemical recurrence status. The principal component analysis based on HRMAS results revealed that tissue spermine level could be used to identify PCa recurrence after prostatectomy [68].

It is difficult to separate the individual metabolite signals due to overlapped choline, polyamine (mainly spermine), and citrate spectral lines between 3 and 3.2 ppm using nuclear magnetic resonance spectroscopy (NMR spectroscopy), and the ratio of choline over citrate plus spermine, namely the Cho/(Cit+Spm) ratio, was found to discriminate between PCa and healthy tissue [69], and a higher metabolite ratio of (choline+creatine)/spermine (Cho+Cr/Spm) was shown in higher Gleason scores (4+3) subgroups of PCa compared with lower Gleason scores (3+4) subgroups by (2D) J-resolved NMR [70]. In addition, several other studies revealed that the ratio of (choline plus spermine plus creatine) over citrate, (Cho+Spm+Cr)/Cit, can be used as an indicator of PCa aggressiveness [65,71,72]. Because increased choline level coincides with decreased spermine level in PCa, which may reduce overall sensitivity of the method, but higher field strengths, such as 3T or even 7T can improve its performance in PCa detection [73].

The TMPRSS2-ERG gene fusion is the most common gene rearrangement in PCa which is associated with cancer cell invasion and proliferation [74]. Two independent PCa patient cohorts conducted by HRMAS revealed lower concentrations of spermine in ERG_high_ patients compared to ERG_low_ samples, indicating an increased cancer aggressiveness of ERG_high_ compared to ERG_low_. Further polyamine pathway study revealed that increased SRS and SSAT in ERG_high_ samples is one of the reasons for a lower spermine level in ERG_high_, which can lead to rapid spermine consumption in cancer cells [75]. As for the prognosis value of spermine in PCa, Braadland et al. showed that decreased spermine concentration can be identified as an independent prognostic marker with shorter recurrence-free survival using ex vivo HRMAS on tissue samples from 110 PCa patients treated with radical prostatectomy [76].

A study by Amita et al. using immunofluorescence further confirmed that spermine content was significantly lower in high-grade prostate intraepithelial neoplasia (HGPIN) and PCa tissues. Enzymes of spermine metabolism pathway (ODC, PAO and SMS) showed opposite expression levels, with significantly higher level in HGPIN and PCa tissues [62]. Prostatic secretory granules (PSG), present in the secretory cells of the glandular prostate, are the major secretory pathway of the prostate gland [77]. Cohen et al. found that PSG consumption during the development of normal prostate epithelial cells through dysplasia to adenocarcinoma was accompanied by reduced spermine. The decreased spermine expression in untreated PCa is linked to PSG loss, and furthermore, androgen deprivation therapy can prevent spermine production in normal prostate secretory cells and paralleled PSG depletion, but spermine will continue to be produced in androgen-resistant tumor clones [78].

### 4.3. Human Expressed Prostatic Secretions (EPS)

Apart from direct monitoring of prostate tissues, prostatic fluid collected after prostate massage is richer in prostatic metabolites, and is less affected by confounding factors [79]. There were two reports suggested an association between spermine concentration in EPS and PCa, both of which were detected by ^1^H-MRS method (Table 1). They found that spermine concentration in human EPS can act as a potential marker of PCa that is independent of age, and decreased spermine level was highly predictive of PCa and negatively correlated with the risk of PCa [80,81].

### 4.4. Erythrocyte

Greater than 95% of circulating spermine and spermidine are transported by erythrocyte [82]. A total of four papers reported “erythrocyte spermine and PCa”, and all of them were published by the same research team [83,84,85,86] (Table 1). In 1990, Cipolla et al. found that erythrocyte spermine levels were significantly correlated with PCa stages, with a higher level in metastatic PCa and hormone-refractory PCa patients [83]. Later, further study was conducted by comparing two cohorts of hormone-treated PCa patients with or without progression, results revealed that increased pretherapeutic erythrocyte spermine level in patients with a higher risk of PCa progression/recurrence, which may help distinguish those people who may benefit more from aggressive primary treatments [84]. In addition, they found that elevated erythrocyte spermine level was an independent prognostic variable for shorter progression-free survival and cancer-special survival in PCa patients [85]. As for metastatic PCa patients, pretherapeutic erythrocyte spermine level also has a significant prognosis and hormonal escape prediction value, which can help discriminate risk of PCa recurrence after hormone treatment [86].

## 5. Therapeutic Potential for PCa by Targeting Spermine Metabolism Pathway

Genetic and epigenetic changes can be heterogeneous, so targeting the metabolic phenotype of cancer that is downstream of multiple common genetic changes may provide a new and effective treatment perspective [87]. The prostate has the highest level of spermine biosynthesis than any other organ, and spermine homeostasis plays an important role in the prostate, thus tumor tissues derived from this gland may have a regulatory response to spermine metabolism pathway [5].

### 5.1. Inhibition of Anabolism Pathway

Increased activity of polyamine metabolism enzymes has been reported in PCa, especially ODC and AMD, which are the first and second rate-limiting enzymes in spermine biosynthesis pathway, respectively [62,88]. A variety of strategies targeting these two key biosynthetic enzymes have been validated as drug targets for PCa in cell and animal models, as well as various clinical trials (Table 2). Some competitive inhibitors of AMD, such as MGBG and CGP-48664, had revealed a potent antiproliferative activity in vitro and in vivo [5,89,90,91]. Difluoromethylornithine (DFMO), the most widely studied ODC inhibitor, has significant inhibitory effects on the growth of cultured PCa cells and animal models [89,92,93,94]. However, in some clinical trials, DFMO, either alone or in combination with other chemotherapies, was generally found to have little antitumor activity [95,96,97]. The limited antitumor activity of DFMO is partly due to a compensatory extracellular polyamine uptake mechanism upon the occurrence of depleted polyamine pools [98]. In contrast to the rather moderate effect of DFMO on models with established cancer, there is increasing interest in using DFMO as a potential strategy for cancer chemoprevention. Studies showed that normal epithelial prostate cells overexpressing ODC undergone malignant transformation in vitro and in vivo [62]. The use of DFMO could suppress the chemically induced prostate carcinogenesis in transgenic adenocarcinoma mouse prostate (TRAMP) models [99]. In addition, a clinical study by Simoneau et al. also showed that DFMO treatment was associated with decreased prostate growth in healthy men with a family history of PCa [100] (Table 2).

### 5.2. Catabolism Pathway Activation

As an alternative to blocking biosynthesis, activation of spermine catabolism by inducing the rate-limiting enzyme SSAT may offer distinct advantages. Studies by Kee et al. found that SSAT induction in cancer cell and TRAMP mice led to PCa growth inhibition in vitro and in vivo. The effect was not attributed to polyamine pool depletion, but by a heightened metabolic flux [101,102]. A possible explanation for the growth inhibition caused by SSAT overexpression may be attributed to the accelerated metabolic flux, causing excessive consumption of important metabolites, such as acetyl-CoA and SAM, and the production of potentially harmful compounds, such as hydrogen peroxide and reactive aldehydes [101].

### 5.3. Development and Use of Polyamine Analogues

In addition to the development of drugs that target specific enzymes in spermine metabolism pathway, another promising option is the application of synthetic polyamine analogues, which have been evaluated to produce a significant PCa growth inhibition effect in vitro and in vivo (Table 2). Symmetrically substituted bis(ethyl) analogues of spermine, i.e., BE-3-3-3 (also known as DENSpm), BE-4-4-4-4 and BE-3-7-3, nonsymmetrically substituted alkylated analogues, CPE-3-3-3, CHE-3-3-3 and the spermine analogue 1,12-diaziridinyl-4,9-diazadodecane (BIS), all have been tested in a variety of human PCa cell lines [5,49,51,103,104,105,106,107] (Table 2). From these studies it can be concluded that these analogues have varied effects in different PCa cells. For example, the androgen-independent DU-145 cells were the most sensitive, whereas the well-differentiated androgen-dependent LNCaP cells were relatively insensitive, which indicate that these analogues might have chemotherapeutic potential for PCa that has failed hormone therapy. The effect of BE-3-3-3, BE-4-4-4-4 and BIS were also examined in the nude mouse xenografts derived from DU-145, PC-3 or DuPro-1 cells with different degrees of malignancy, and results revealed an inhibitory effect on tumor growth, as well as decreased spermine levels [49,103,104]. Moreover, polyamine analogues in other types of conformations, such as macrocyclic, long-chain, and cyclopropane-containing analogues, could also have an inhibitory effect on PCa growth [108,109,110].

## 6. Conclusions and Future Directions

Prostate has a large number of glandular epithelial cells that form lumina, where secreted products such as spermine is concentrated. While an increased level of the other two polyamines, putrescine and spermidine, were observed for cell proliferation, spermine play a more significant role in affecting the function and secretion of prostate epithelium. Studies demonstrated a high level of spermine in normal and BPH tissues but a lower level in PCa tissues and more advanced PCa in particular, indicating spermine as an indicator of malignant phenotype transformation. Urinary spermine level was also found to be significantly reduced in PCa and could serve as a potential biomarker for noninvasive diagnosis of PCa. The exact mechanism of decreased spermine level in PCa is still unclear, but increased SMO and SSAT expression leading to a depletion of spermine content may be implicated. Although early studies demonstrated some potential in PCa treatment by targeting spermine metabolism pathway, further studies are required to elucidate the exact mechanism of spermine in PCa, the diagnostic role of spermine in aggressive PCa, and the potential therapeutic value by targeting spermine pathway in PCa.

## Figures and Tables

**Table 1:** Spermine studies performed in urine/tissues/EPS/erythrocyte from PCa patients.

Publication	Sample Type	Analytical Platform	PCa Group	Control Group	Main Results	Statistical Significance	Ref.
Sanford et al., 1975	24-h urine	Beckman spectrophot-ometer	*n* = 15 PCa	*n* = 42 healthy controls	↑Polyamines in 11/15 PCa	NR	[56]
Fair et al., 1975	12-h/ 24-h urine	Spectronic 20 colorimeter	*n* = 44 PCa	*n* = 13 healthy controls	Similarly low levels of spermine detected in cancer and healthy controls	NR	[57]
Sugimoto et al., 1995	Morning Urine	HPLC	*n* = 24 urogenital cancer, including 13 PCa	*n* = 43 benign urogenital disorders; *n* = 52 healthy controls	↑DiAcSpm in urogenital cancer	NR	[58]
Hiramatsu et al., 1997	Morning Urine	HPLC	*n* = 31 urogenital cancer, including 15 PCa	*n* = 42 benign urogenital disorders; *n* = 52 healthy controls	↑DiAcSpm in urogenital cancer ↑DiAcSpm in cancer patients with poor prognosis and recurrence	NR	[59]
Tsoi et al., 2016	Pre-biopsy urine with serum PSA level >4.0 ng/mL	UPLC–MS/MS	*n* = 66 PCa	*n* = 88 BPH, *n* = 11 healthy controls	↓Spermine in PCa; AUC of spermine for PCa: 0.83	*p* < 0.0001	[60]
Chiu et al., 2021	Pre-biopsy urine with serum PSA level 4–20 ng/mL	UPLC–MS/MS	*n* = 185 PCa; *n* = 103 HGPCa	*n* = 415 healthy controls	↓Spermine in PCa and HGPCa; AUC of spermine risk score: PCa 0.78, HGPCa 0.82	*p* < 0.001	[61]
Graaf et al., 2000	Tissue	HPLC	*n* = 7 PCa	*n* = 4 healthy controls, *n* = 3 BPH	↓Spermine in PCa	*p* < 0.05	[7]
Swanson et al., 2003	Tissue	HRMAS	*n* = 7 PCa (gland percentage < 20) *n* = 13 PCa (gland percentage ≥ 20, 8 with GS ≤ 6, 5 with GS ≥ 7)	*n* = 33 healthy controls	↓Spermine in PCa compared with controls ↓Spermine in PCa with higher GS	*p* = 0.01 *p* = 0.05	[64]
Swanson et al., 2006	Tissue	HRMAS	*n* = 60 PCa	*n* = 6 healthy controls	↓Spermine in PCa	*p* < 0.01	[66]
Maxeiner et al., 2010	Tissue	HRMAS	*n* = 16 PCa with BCR	*n* = 32 PCa without BCR (16 clinical-stage-matched and 16 pathological-stage-matched)	Spermine alteration predicts PCa recurrence	NR	[68]
Nagarajan et al., 2010	Tissue	(2D) J-resolved spectroscopy (JPRESS)	*n* = 7 PCa with GS = 4 + 3	*n* = 7 PCa with GS = 3 + 4	↑(Cho + Cr)/Spm ratio in PCa with GS = 4 + 3	*p* = 0.07	[70]
García-Martín et al., 2011	Tissue	^1^H-MRS	*n* = 30	*n* = 249	↑Cho/(Cit + Spm) ratio in PCa	*p* < 0.001	[69]
Giskeodegar-d et al., 2013	Tissue	HRMAS	*n* = 30 PCa with GS = 6; *n* = 81 HGPCa with GS ≥ 7	*n* = 47 normal adjacent samples	↓Spermine in PCa and HGPCa compared with normal ↓Spermine in HGPCa compared with PCa ↑(Cho+Spm+Cr/Cit) ratio in HGPCa	*p* = 0.022 *p* = 0.0044 *p* = 2.17 × 10^-4^	[65]
Selnaes et al., 2013	Tissue	In vivo MRSI and ex vivo HRMAS	*n* = 15 PCa with GS ≥ 4 + 3 for ex vivo HRMAS *n* = 19 PCa with GS ≥ 4 + 3 for in vivo MRSI	*n* = 16 PCa with GS ≤ 3 + 4 for ex vivo HRMASn = 12 PCa with (GS ≤ 3 + 4) for in vivo MRSI	↑(Cho+Spm+Cr/Cit) ratio with increasing GS	*p* = 0.035 (ex vivo) *p* = 0.001 (in vivo)	[72]
Basharat et al., 2015	Tissue	HRMAS	*n* = 8 PCa with T3 stage *n* = 19 PCa with GS = 7	*n* = 7 PCa with T1 stage, *n* = 11 with T2 stagen = 6 PCa with GS = 6	↓Spermine in PCa with advanced stage and higher GS	T3 vs. T1 *p* = 0.04 T3 vs. T2 *p* = 0.08 GS = 7 vs. GS = 6 *p* = 0.01	[67]
Hansen et al., 2016	Tissue	HRMAS	*n* = 34 ERG_high_ PCa	*n* = 30 ERG_low_ PCa	↓Spermine in ERG_high_ PCa compared with ERG_low_ PCa	*p* < 0.001	[75]
Shukla-Dave et al., 2016	Tissue	Immunofluo-rescence	*n* = 18 HGPIN; *n* = 120 PCa	*n* = 103 healthy controls	↓Spermine in HGPIN and PCa	*p* < 0.0001	[62]
Braadland et al., 2017	Tissue	HRMAS	*n* = 50 PCa with recurrence	*n* = 60 PCa without recurrence	↑Spermine independently associated with better RFS ↑(Cho+Cr)/Spm independently associated with worse RFS	RFS: HR = 0.72, *p* = 0.016 RFS: HR = 1.43, *p* = 0.014	[76]
Lynch et al., 1997	EPS by prostatic massage	^1^H-MRS	*n* = 4 PCa	*n* = 12 healthy controls; *n* = 10 BPH; *n* = 11 vasal aplasia, *n* = 1 prostatodynia	↓(Cit to Spm) ratio in PCa	*p* < 0.02	[80]
Serkova et al., 2008	EPS by prostatic massage	^1^H-MRS	*n* = 52 PCa	*n* = 26 healthy controls	↓Spermine in PCa	*p* < 0.002	[81]
Cipolla et al., 1990	Erythrocyte spermine	HPLC	*n* = 36 PCa with metastases; *n* = 12 PCa with hormonal escape	*n* = 17 PCa without metastases; *n* = 41 PCa with hormonal responsiveness	↑Spermine in PCa with metastases ↑Spermine in hormone-refractory PCa	*p* < 0.01 *p* < 0.001	[83]
Cipolla et al., 1993	Erythrocyte spermine	HPLC	*n* = 28 endocrine-treated PCa with progression	*n* = 23 endocrine-treated PCa without progression	↑Pretherapeutic spermine level in PCa with progression	*p* < 0.01	[84]
Cipolla et al., 1994	Erythrocyte spermine	HPLC	*n* = 40 newly diagnosed, stage D2 PCa	NA	↑Spermine associated with shorter PFS and CSS in PCa	PFS: *p* = 0.001 CSS: *p* = 0.0025	[85]
Cipolla et al., 1996	Erythrocyte spermine	HPLC	*n* = 88 PCa with metastases	NA	↑Pretherapeutic spermine level predicts worse PFS and CSS in metastatic PCa	PFS: *p* < 0.0001 CSS: *p* < 0.0005	[86]

Abbreviations: NR, not reported; ROC, Receiver operating characteristics; GS, Gleason score; BCR, biochemical recurrence; HGPCa, high-grade prostate cancer; HGPIN, high-grade prostatic intraepithelial neoplasia; RFS: recurrence-free survival; NA, not available; PFS: progression-free survival; CSS: cancer special survival.

**Table 2:** Inhibitors targeting spermine metabolism pathway in cell and animal models, as well as clinical trials for PCa.

	Publication	Inhibitor	Target	PCa Cell Lines	PCa Animal Models	Clinical Trials	Main Results	Ref.
**Polyamine Synthesis Inhibitor**	Heston et al., 1982	DFMO	ODC	R3327 MAT-Lu^a^	Rat injected with R3327 MAT-Lu	NR	In vitro and in vivo: Inhibition of R3327MAT-Lu growth	[92]
	Dunzendorfer et al., 1983	DFMO/MGBG/DFMO + MGBG	ODC + AMD	NR	Rat injected with R3327-G^b^	NR	In vivo: Inhibition of R3327-G growth by either DFMO or MGBG; their combination was more effective	[89]
	Herr et al., 1984	DFMO/MGBG/DFMO + MGBG	ODC/AMD/ ODC + AMD	NR	Rat injected with R3327-G	NR	In vivo: DFMO had no antitumor effect, MGBG retarded tumor growth; their combination inhibited tumor growth	[90]
	Herr et al., 1986	DFMO + MGBG	ODC + AMD	NR	NR	Phase I; 5 advanced, hormone-resistant PCa patients	Clinical trial: No antitumor effects, but drugs were well tolerated	[95]
	Horn et al., 1987	DFMO ± doxorubicin + cyclophosphamide	ODC ± conventional chemotherapy	NR	NR	Phase I-II; 9 PCa patients (DFMO + conventional chemotherapy); 5 PCa patients (conventional chemotherapy)	Clinical trial: No effect	[96]
	Kadmon et al., 1992	DFMO	ODC	NR	Rat injected with R3327 MAT-Lu	NR	In vivo: Modest inhibition of R3327 MAT-Lu growth	[93]
	Delworth et al., 1995	CGP-48664	AMD	LNCaP, LNCaP-LN3, PC-3M, and PC-3M-MM2	Nude mice injected with LNCaP-LN3 cells or PC-3M-MM2 cells	NR	In vitro and in vivo: Induction of cytostasis; Inhibition of tumor growth in slow-growing tumor, but not fast-growing tumor	[91]
	Mi et al., 1998	CGP-48664	AMD	LNCaP, DU145, and PC-3	NR	NR	In vitro: Inhibition of PCa cells growth	[5]
	Carbone et al., 1998	DFMO/PXM/ DFMO + PXM	ODC/ prostaglandin Inhibitor/ODC + prostaglandin Inhibitor	NR	NR	Phase I; 31 cancer patients, including stage A or B PCa	Clinical trial: Dosage toxicity assessment	[97]
	Gupta et al., 2000	DFMO	ODC	NR	TRAMP	NR	In vivo: DFMO prevent prostate tumorigenesis	[99]
	Devens et al., 2000	DFMO	ODC	PC3, LNCaP, DU145	Nude mice injected with PC-3 cells	NR	In vitro and in vivo: Inhibition of tumor growth	[94]
	Simoneau et al., 2008	DFMO/ placebo	ODC	NR	NR	81 men with PCa family history but without personal PCa history	Clinical trial: Induction of slow prostate growth and no grade 3 or 4 toxicities	[100]
**Polyamine Analogues**	Jeffers et al., 1997	BE-4-4-4-4/BE-3-7-3/BE-3-3-3	PA analogue	DU145, LNCaP and PC-3	BALB/c mice injected with DU145 cells	NR	In vitro and in vivo: Inhibition of PCa cells growth by all three polyamine analogues	[103]
	Zagaj et al., 1998	BE-4-4-4-4	PA analogue	AT3.1, AT6.1 and AT6.3; DU145, DuPro-1 and TSU-Pr1	Nude mice injected with DuPro-1 and PC-3 PCa cells	NR	In vitro and in vivo: BE-4-4-4-4 was cytotoxic against rat and human PCa cells	[104]
	Mi et al., 1998	DENSpm	PA analogue	LNCaP, DU145, and PC-3	NR	NR	In vitro: Inhibition of PCa cells growth with varied sensitivity, DU145 > PC-3 > LNCaP	[5]
	Eiseman et al., 1998	BIS	PA analogue	PC-3 and DU-145	Nude mice injected with PC-3 and DU-145 cells	NR	In vitro and in vivo: Inhibition of PCa growth and enhanced radiosensitivity	[51]
	Schipper et al., 2000	DENSpm	PA analogue	PC-3, TSU-pr1, DU-145, and JCA-1	Nude mice injected with Du145 cells	NR	In vitro and in vivo: Inhibition of PCa growth	[49]
	McCloskey et al., 2000	CPENSpm, CHENSpm and BE 3-3-3	PA analogue	LNCaP, PC3, and Du145	NR	NR	In vitro: Inhibition of PCa cells growth, especially DU145	[105]
	Reddy et al., 2001	BE-4-4-4-4	PA analogue	LnCap, DU145, PC-3, and DuPro	NR	NR	In vitro: Inhibition of PCa cells growth with varied sensitivity, LnCap and DU145 > DuPro >PC-3	[107]
	Valasinas et al., 2001	BE-4-4-4-4	PA analogue	LnCap, DU145, DuPro, and PC-3	NR	NR	In vitro: Inhibition of PCa cells growth, PC3 was the least sensitive	[106]
	Frydman et al., 2003	Cyclopropane-Containing Polyamine Analogues	PA analogue	DU-145, DuPro, and PC-3	NR	NR	In vitro: Inhibition of PCa cells growth, especially DU145	[110]
	Valasinas et al., 2003	Long-chain Polyamines (Oligoamines)	PA analogue	LnCap, DU-145, DuPro and PC-3	NR	NR	In vitro: Inhibition of PCa cells growth with varied sensitivity, LnCaP, DU145 > DuPro and PC-3	[109]
	Frydman et al., 2004	Macrocyclic Polyamines	PA analogue	DuPro and PC-3	NR	NR	In vitro: The macrocycles were cytotoxic against PCa cells	[108]

^a^ R3327MAT-Lu, metastatic derivative of the Dunning slow-growing, androgen-responsive prostatic adenocarcinoma; ^b^ R3327-G, G subline of the Dunning rat that is a poorly differentiated PCa cell lines. Abbreviations: NR, not reported; TRAMP: Transgenic adenocarcinoma mouse prostate.

## Data Availability

Not applicable.
